# The Practice of Surgery: Embracing Minor Surgery and the Application of Dressings, &c., &c., &c.

**Published:** 1849-11

**Authors:** 


*The Practice of Surgery: embracing Minor Surgery and the Application of Dressings, &c., &c., &c.* By JOHN HASTINGS, M. D., U. S. N., Fellow of the College of Physicians of Philadelphia, Member of the Philadelphia Medical Society, Lecturer on Surgical Anatomy and Operative Surgery, &c., &c., &c. With numerous Illustrations. Philadelphia: Lindsay & Blakiston, 1850. 8vo. pp. 479.

This volume forms one of a series of books now in process of publication by Messrs. Lindsay & Blakiston. Three have been already issued from the press, embracing, respectively, Midwifery, the Elements of General Pathology, and the Diseases of Children. Of the character of these works we have formed an exceedingly favorable opinion, believing that they have been written with judgment, and with fidelity to what can reasonably be considered the truths of Medical Science, touching the branches of which they severally treat. They do not contain all the theories and opinions prevalent at the present day concerning these subjects; the character and intention of the publications would not have admitted of this. Their object was to present to medical students and to young practitioners of medicine, chiefly, a carefully prepared digest of the Principles and Practice of Medicine, as connected with the above mentioned departments, so far as principles and practice can be considered as well ascertained and trustworthy. This object has, we think, been well attained; and if the subsequent treatises be equally good, we think, further, that their authors, their readers, and the publishers will have reason to feel pleased and satisfied.

Writing, be it never so creditably accomplished, is a very thankless task, excepting to some few happy ones who, in addition to the ability to write, and sometimes without it, possess the "imprimatur" which high professional position affixes to their labors. Every thing which their pens may indite, is at once met, at more than half-way, with a welcome, and a conviction of its great worth; while it is generally assumed, on the other hand, that young men, who have not yet attained to the gown and the chair of the professorial dignity, or to the charge of hospitals, know nothing, and can communicate less, that is worth hearing or reading. These are prejudged to be guilty of ignorance, and are required to prove their innocence against " a great cloud of witnesses,"—cold friends, jealous rivals, scornful superiors, and censorious critics, who, like the horrible prowlers around a field of battle, grow rich by despoiling those whom they help to murder. Surrounded thus by natural difficulties, and by obstacles heaped in their path, they have an unequal contest to maintain: like Ajax amidst the darkness sent from heaven, and the hosts of Troy, they must fight no longer for glory, but for very life. We think that this is unfair and uncharitable. We honor youthful ambition and youthful valor, and bid them God-speed; if we must fight, we would rather oppose the " vieux moustaches."

The author of the volume, of which the title is prefixed to this article, is known to us as a zealous and successful student of anatomy, and to have had opportunities of gaining large experience, as a surgeon, in the naval and civil services. We propose, briefly, to examine the contribution which he has just made to " The Medical Practitioners' and Students' Library."

The author's object has been " not to confuse his readers with a laborious attempt to embrace each detail of fact and theory, but rather to point out only the most prominent and distinctive features of a very fertile subject. Under this restricted plan, he hopes that his treatise may serve as a *guide-book* to the *student* and *young practitioner of surgery.*" The book is divided into five parts. The *first division* treats of Inflammation and its varieties; Suppuration and Abscess when localized in its most favorite regions; wounds and their effects, including Hydrophobia and Tetanus. The *second* describes the methods of performing the operations—the simple and the more important—which are practised upon the various regions and organs of the body. The *third* is devoted to the consideration of Fractures and Dislocations. The *fourth* discusses the injuries and surgical diseases to which the individual regions of the body are liable, apart from those embraced under the preceding divisions, and others treated of under the *fifth* and last, which are thus enumerated: " diseases of the Arteries, injuries and diseases of the Veins, injuries and diseases of the Nerves, Venereal disease, diseases of the Bones, malignant tumors of Bone, diseases and injuries of Joints, injuries and diseases of the Ear, injuries and diseases of the Eye." It will thus be perceived, that the author has discussed a vast number and variety of topics; and it is a matter of astonishment to us that he has been able to treat satisfactorily of all, within such restricted limits.

At the very outset of his labors, Dr. Hastings must have been much perplexed to determine what plan he could adopt which would embrace all the subjects of which he was expected to treat, and by which he might secure simplicity, harmony and perspicuity of arrangement, and, at the same time, avoid repetition. We do not think of any one, at this moment, by which all these desiderata could be attained. It seems to us, however, that the arrangement which he has followed is faulty as regards convenience of reference, since in very many instances the mode of treatment recommended for diseases and injuries is to be sought for under another division of the book than that which describes these affections. Thus, Hernia, Fistula in ano, Phimosis, the presence of foreign bodies in the Larynx and Trachea, are treated of in various sections of the fourth division of the volume, while the operations necessary for their relief are discussed in the second. It would be better, we think, that the remedy should be more immediately associated with the description of the disease, and that the division devoted to operations should include only those generally associated with Minor Surgery, the ligature of Arteries, and Amputations. Again, the diseases of the Mammæ are described in two distinct sections of the fourth division, separated from each other by *ten* intervening chapters on different diseases and injuries; and, moreover, in each of these two sections the same diseases are, in several instances, spoken of.

Our limits will not permit us to review the manner in which the author has treated of all the subjects comprised in his volume; we must content ourselves with noticing a few of the more important.

Inflammation first occupies his attention, and to this subject twenty-four pages or more are devoted. He objects to what are usually considered "the terminations" of inflammation; and we think that the ground of his objection is a very just one—that the secondary *results* of this process are very generally confounded with its real *terminations*. We *infer* from the author's remarks, although he does not so state them with perfect distinctness, that he allows but *three* terminations of inflammation, viz., resolution, effusion, and mortification. We agree with the author in this opinion; yet it seems to us that his reasoning on the subject is not very clear, and involves some contradictions and some errors. We desire, however, to speak with diffidence; for inflammation has always appeared to us a process difficult to be understood, partly from its complex character, and partly in consequence of the conflicting and contradictory statements of facts and conjectures concerning it. It is apparently easy to ascertain the causes which most frequently excite it, and to account for the most striking of its sensible phenomena; but its real nature is still a mystery. In attempting to unravel this, it seems to us that too much importance is attached to the operation of merely physical laws and conditions. Thus, the effusions which occur are generally ascribed to the effect of pressure, from the continuance of the heart's action, upon a more or less stagnant fluid which distends the capillaries and arteries of the inflamed part; the influence of modified vital action upon the coats of these vessels is rarely appealed to, yet such modified action must exist, and there is great reason to believe that this will more rationally account for the escape of the fluids usually effused in inflammation; (see Experiments by Sir John Leslie, quoted in a note to p. 802, vol. 5, of the London Med. Gaz. for 1847;) the arrest, retardation, or acceleration of the flow of the blood, and the dilatation or contraction of the vessels, seem to engage the whole attention.

We suppose that inflammation is always a salutary process in its object; we cannot in every case see this motive clearly, but generally it is, to furnish the materials for the restoration of lost tissue, as after a contused wound; or to prevent the extension of some destructive action, as of tubercular ulceration of the lung, and subsequent gangrene of the pleura; or to furnish a foreign substance, accidentally or otherwise lodged in the tissue, with an enveloping membrane which eventually becomes a part of the tissue, and thus removes from the intruding object its power to harm; or, finally, to extrude a foreign body. This last may furnish us with an illustration of the process of inflammation. A splinter becomes lodged in the finger; speedily the latent preservative powers with which the whole system and each part of it are endowed, are aroused by the irritation of this noxious agent; the first impression may be made upon the nerves of the part, but soon all the associated elements of the inflammatory process are in operation, for the purpose of effecting the complete removal of the disturbing cause. This object is accomplished first by the effusion of fibrin around it; and secondly by the conversion of this fibrin into pus, the escape of the latter, and, with it, of the splinter. It may or may not be, as Dr. Hastings supposes, essential to constitute inflammation that "the circulation be entirely arrested in the disordered part," p. 36. We presume that this is not always the case, and that in most instances the circulation is "partly increased, partly diminished;" (Williams' Princip. Med.) but in all local inflammations, this condition is less important than the character of the effusion, and the changes which take place in it. The author's views seem to be confused and contradictory on these points: thus, he says, page 33, "there is now inflammation of the different tissues, and gradually approaching *death of the effused fibrin*," &c.; and again, at page 35, he says of the products of inflammation, that they "*all become foreign and effete bodies*," &c.; yet he acknowledges that fibrin is the material "to which the reparative operations of Nature are due," p. 31. How can a living tissue be made from a substance which is dead and fit only to be thrown off? The fibrin effused in healthy inflammation is not an effete matter, but a living substance, which either becomes incorporated with and assimilated to the tissue into which it has been extravasated, or undergoes certain other vital changes. In either event the purpose is a salvatory one; in the first, the disturbing cause is thereby removed, and the preservation of the part is secured; by the last these objects are placed in process of gradual attainment: the illustration before commenced, will continue to explain this position. The splinter is surrounded by effused fibrin; but the former is an inert foreign body, incapable of withdrawing itself from the tissue, or of being removed by any force or action of its own; and the tendency of effused fibrin is to become organized, and to be a living part of the living tissue, not to leave this. Still the splinter must be thrown off. How? Simply through the influence of a vital change effected in the fibrin which enwraps it; the fibrin is converted into pus, of which the peculiar tendency is of itself to escape by external rejection. We cannot allow ourselves the space necessary to attempt an explanation of the modus operandi of this conversion of the plasma into pus, and the escape of the latter; we will refer the reader to Vogel's Pathological Anatomy, as affording an exceedingly interesting and satisfactory dissertation on the subject, and also to the article on inflammation and its results, by the same author, in Wagner's Physiology and Pathology.

Dr. Hastings' opinions concerning the origin and character of pus, —that it "is probably nothing but dead red corpuscles and exudation corpuscles floating in an altered serum, which holds in solution other ingredients of the blood," p. 34,—are, we think, untenable, if we may rely upon the examinations of careful microscopists, (see Vogel, Henle, &c. &c.) These views also lead him to assimilate suppuration with mortification, (see "Mortification," p. 37.) We dissent also from the statements made with reference to the possibility of distinguishing the pus-corpuscle, under the microscope, p. 61: according to the evidence of Vogel and other reliable micrographers, the true pus-globule may always be recognized by its shape and size, as viewed with the microscope, and by the peculiarity of its nucleus which, when the globule is treated with acetic acid, is seen to be "generally composed of several minute granules forming a composite multiple nucleus." Vogel, p. 132.

With the author's views concerning the *treatment* of inflammation, we have been well pleased. He suggests nothing novel, but he has the greater merit of stating clearly and with judgment those facts which are already known. We would particularize that the remarks on the local treatment, so far as the application of cold and warm dressings is involved, the circumstances which should prompt to the preference of one rather than of the other, and to the substitution of one for the other, are very judicious.

We pass to the chapter on the Operations. Those which are employed for the relief of Hernia, are among the most important, and are the only ones which we shall be able to notice. In the description of these, we are struck with the observation of many inaccuracies and vaguenesses of style and expression, which we attribute, of course, to inadvertence on the author's part; and yet they may give rise to confusion. We will mention some of these. In the definition of "Taxis," the relaxation of the muscles is included, as well as the manual operation; in detailing the direction in which the taxis should be made to restore an oblique inguinal hernia, it is stated that "the pressure must be applied upwards an l *downwards* to force the protrusions into the canal," p. 88 ; this is really correct, as the patient is supposed to be lying on his back, and the pressure *should* be made in the direction upwards, with reference to his head, and downwards, with reference to the cavity of the abdomen ; yet we think that upwards and *inwards*, or upwards and *backwards*, would be a more intelligible expression ; continuing this subject,—the restoration of an oblique inguinal hernia,—the text says, the pressure "should then be obliquely outwards, upwards and downwards," &c. ; we would suggest the substitution of the same word as before for "*downwards*."

The author recommends that, the "taxis should be repeated, at intervals of one or two hours, until it succeeds, or until all reasonable hope of success is at an end ; which is the case when the hernia has been strangulated for about thirty-six hours, or very little beyond this time, for mortification of the intestine occurs rapidly." p. 88. The adjuvants to the taxis are stated, though with scarcely sufficient force, considering their importance ; and in the mention of *ice* as one of these, it is recommended, not for the purpose of assisting in the reduction of the hernia, but "to retard the advance of inflammation." We think that it is injudicious to prescribe any number of hours during which it may be safe to postpone the operation in the event of the failure of the taxis, bleeding, the warm bath, the application of cold, the use of tobacco clysters ; it is much safer to be guided by the condition of the individual patient ; Sir A. Cooper's advice is strong upon this point.

The proper mode of using the knife in this operation is very well expressed ; yet here, also, a little more clearness and harmony in the arrangement of the descriptions of its different steps is desirable. (The author does not always keep in mind that an explanation which would be sufficiently transparent to himself, and others equally advanced, might be cloudy to a student.) In endeavoring to ascertain the seat of the *stricture*, the author advocates the proper method ; but he errs, we think, in the opinion that, the same exploration which enables the finger to detect the point of strangulation will permit of the formation of a decision, as to whether the hernia be oblique or direct, by fixing the position of the epigastric artery. For suppose the stricture to be at the external ring; in order that the finger shall be able to reach the epigastric artery, which is at some distance from the ring, the stricture must first be passed, yet a stricture which would admit of this would be no stricture. This must be simply an error of expression, however, on the author's part.

In enumerating the possible points of stricture in *femoral* hernia, the author mentions the falsiform process of the fascia femoris, and Hey's ligament, as though they were different structures, and not synonymous with each other.

It would have been well had the author prefixed to the description of these operations, a brief account of the Anatomy of the parts, in which the relative situation of the arteries concerned had been particularized.

With regard to the other operations, we will only say that the author has been judicious in his selections, and as liberal in his details as his limits would permit; it is impossible that, in so small a book, every operation can be described, and it is necessary that a large discretion should be allowed to the author, under these circumstances.

On the important subjects of Fractures and Dislocations, the chapters are full and interesting, and contain many valuable hints and recommendations, some of which we do not remember to have elsewhere met with. The author evidently speaks from experience in these matters, and his remarks commend themselves at once to the reader.

We can speak generally in similarly commendatory terms of the chapters devoted to the consideration of the various diseases and injuries of different organs. A brief, clear and decided exposition of the phenomena and treatment of disease is, on many accounts, and in many circumstances, much more desirable than a very copious array of opinions and rules. The dissertation on *Aneurism*, however, should have been more full and complete, and the author should not have failed to dwell emphatically on the treatment by pressure, so successfully practised by some of the Irish surgeons and others. The chapters on *Venereal diseases* are much more satisfactory, and will interest and instruct. He recommends strongly a cold infusion of the powdered root of "the Hydrastis

Canadensis, called Golden Seal," in the proportion of ℥ss. to ½ pint of cold water, as an injection in gonorrhœa.

On the subject of the diseases of *the Eye*, the author's chief authorities are Sichel and Weller. We have long been fond of Sichel's book, and have found his descriptions very clear and simple. His treatise is not so generally esteemed in this country as those of the English Ophthalmologists; but it has afforded us more satisfaction, and we are pleased to find that Dr. Hastings appeals to him.

We must now take leave of the author. We have been obliged to differ from him in many instances, but we trust that we have not failed to express our satisfaction in others; "naught did we in hate, but all in honor," and we sincerely hope that there will be speedy occasion for a second edition of his book, which, by being rendered more elegant and precise in style, and further improved by additional time and study bestowed upon its arrangement and preparation, will enhance still more the reputation of the author. F. W. S.

